# Role of inflammatory cytokine in mediating the effect of plasma lipidome on epilepsy: a mediation Mendelian randomization study

**DOI:** 10.3389/fneur.2024.1388920

**Published:** 2024-05-30

**Authors:** Xiangyi Wang, Wenting Xiong, Man Li, Luyan Wu, Yuying Zhang, Chaofeng Zhu, Wanhui Lin, Shenggen Chen, Huapin Huang

**Affiliations:** ^1^Department of Neurology, Fujian Medical University Union Hospital, Fuzhou, China; ^2^Fujian Key Laboratory of Molecular Neurology, Fuzhou, China; ^3^Department of Geriatrics, Fujian Medical University Union Hospital, Fuzhou, China

**Keywords:** epilepsy, Phosphatidylcholine (18:1_18:1), TNFSF12, causal relationship, MR analysis, mediation MR analysis

## Abstract

**Background:**

Epilepsy is one of the most prevalent serious brain disorders globally, impacting over 70 million individuals. Observational studies have increasingly recognized the impact of plasma lipidome on epilepsy. However, establishing a direct causal link between plasma lipidome and epilepsy remains elusive due to inherent confounders and the complexities of reverse causality. This study aims to investigate the causal relationship between specific plasma lipidome and epilepsy, along with their intermediary mediators.

**Methods:**

We conducted a two-sample Mendelian randomization (MR) and mediation MR analysis to evaluate the causal effects of 179 plasma lipidomes and epilepsy, with a focus on the inflammatory cytokine as a potential mediator based on the genome-wide association study. The primary methodological approach utilized inverse variance weighting, complemented by a range of other estimators. A set of sensitivity analyses, including Cochran’s *Q* test, *I*^2^ statistics, MR-Egger intercept test, MR-PRESSO global test and leave-one-out sensitivity analyses was performed to assess the robustness, heterogeneity and horizontal pleiotropy of results.

**Results:**

Our findings revealed a positive correlation between Phosphatidylcholine (18:1_18:1) levels with epilepsy risk (OR = 1.105, 95% CI: 1.036–1.178, *p* = 0.002). Notably, our mediation MR results propose Tumor necrosis factor ligand superfamily member 12 levels (TNFSF12) as a mediator of the relationship between Phosphatidylcholine (18,1_18:1) levels and epilepsy risk, explaining a mediation proportion of 4.58% [mediation effect: (*b* = 0.00455, 95% CI: −0.00120-0.01030), *Z* = 1.552].

**Conclusion:**

Our research confirms a genetic causal relationship between Phosphatidylcholine (18:1_18:1) levels and epilepsy, emphasizing the potential mediating role of TNFSF12 and provide valuable insights for future clinical investigations into epilepsy.

## 1 Introduction

Epilepsy is a chronic central nervous system disease that affects people of all ages, impacting over 70 million people globally (1). It is characterized by persistent seizures originating from abnormal neuronal discharges in the brain, exhibiting paroxysmal, transient, repetitive and stereotyped features (2). Studies have shown that approximately 51 million people worldwide are actively affected by epilepsy as of February 24, 2023 and around 4.9 million new cases emerge annually based on global population and epilepsy incidence rates (3). According to the WHO’s 2010 Global Burden of Disease study, epilepsy is ranked as the second most burdensome neurological disorder worldwide in terms of disability-adjusted life years (4). Despite studies indicating seizure control in over 2/3 patients using antiepileptic drugs, approximately 1/3 develop drug-resistant epilepsy (5). The pathogenesis of epilepsy is complex, often resulting from the combined effect of multiple factors. Age, family history of epilepsy, febrile convulsions, preterm birth, alcohol consumption, central nervous system infection, traumatic brain injury, stroke and sleep deprivation are well-established risk factors for epilepsy (6, 7). However, these traditional risk factors can only partially explain the risk of epilepsy. Therefore, accurately identifying novel epilepsy-related risk factors has become crucial for exploring new therapeutic targets and achieving early diagnosis and treatment.

Recent studies increasingly unveil metabolic or lipid profiles distinguishing responders from nonresponders to anti-seizure medications (ASMs), yet their intricate role in epilepsy remains largely obscure due to complexity (8–10). Lipidomics is an emerging discipline dedicated to the systematic examination of lipids in biological systems, encompassing various fatty acids (FAs) and lipid subclasses. An integrated metabolomics and lipidomics investigation of children with drug-refractory epilepsy (DRE) revealed decreased plasma fatty acids but elevated triglycerides (8). An investigation into kainic acid (KA)-induced acute epilepsy models in mice delineated alterations in phospholipid, endocannabinoid, endocannabinoid-like molecules, arachidonic acid and eicosanoid levels in the brain and periphery (11). Subsequent animal research demonstrated that subchronic administration of palmitoylethanolamide markedly diminished seizure intensity and fostered neuroprotection, which can also modulate plasma and hippocampal endocannabinoid and eicosanoid levels in systemic KA-injected mice (12). An investigation into metabolomic alterations in adult status epilepticus (SE) patients showed that metabolic dysregulation primarily impacted amino acid metabolism, pyrimidine metabolism and lipid homeostasis (13). The existing association between epilepsy and plasma liposomes primarily arises from observational studies characterized by limited sample sizes and numerous confounding variables. The nature of these studies makes it difficult to rule out the possibility of reverse causality, thereby compromising their reliability in establishing causality. In essence, while the correlation between epilepsy and plasma liposomes is evident, the causal relationship remains ambiguous, necessitating further research to elucidate the intricate pathophysiological mechanisms.

Inflammatory cytokines play an important role in infection and inflammation, which serve as crucial mediators for communication between the brain and the nervous system, contributing significantly to epilepsy (14). Neuroinflammation serves as a dual-edged sword, offering protection to the central nervous system as an immune response yet becoming detrimental when overactivated. TNF- *α* are the main inflammatory factors in the pathogenesis of epilepsy (15). In neurological disorders, including epilepsy, excess TNF damages the central nervous system by causing extracellular glutamate to accumulate to levels sufficient to inhibit synaptic activity or kill neurons (16). In a systematic review, the majority of studies corroborate the heightened expression of TNF- α in epilepsy patients, linked to a higher susceptibility to seizures in the future, albeit with inconsistency (15). However, the results of the study regarding the association between inflammatory cytokines and epilepsy lack consistency, warranting further investigation.

Nevertheless, further exploration is warranted to elucidate the specific contribution of plasma lipidome and inflammatory cytokines to epilepsy. Similar to the randomized controlled trial (RCT), the Mendelian randomization (MR) study is an analytical method of epidemiological etiological inference, relying on Mendelian independent distribution to investigate causal relationships between exposure and outcome variables (17). Bayesian weighted Mendelian randomization (BWMR) integrates the statistical methodologies of MR and Bayesian inference to enhance the precision of assessing causal relationships between exposure factors and outcome variables (18). In the MR study, single nucleotide polymorphisms (SNPs) are regarded as an instrumental variables (IVs) to estimate the causal relationship between exposure and outcome (19). Previous studies have found the causal relationship between human blood metabolites, gut microbiota, circulating lipids, psychiatric disorders and epilepsy (20–24). In recent years, an increasing number of studies have examined the association between plasma lipidome and epilepsy, yet no research has explored this relationship at the genetic level. To the best of our knowledge, the causal association between a broad range of plasma lipidome and epilepsy has not been established using MR. This paper conducted a comprehensive mediation MR analysis of plasma lipidome and epilepsy datasets using a large-scale genome-wide association study (GWAS), introducing inflammatory factors as mediators to ascertain the causal relationship between plasma lipidome and epilepsy.

## 2 Materials and methods

### 2.1 Study design

**Figure 1:**
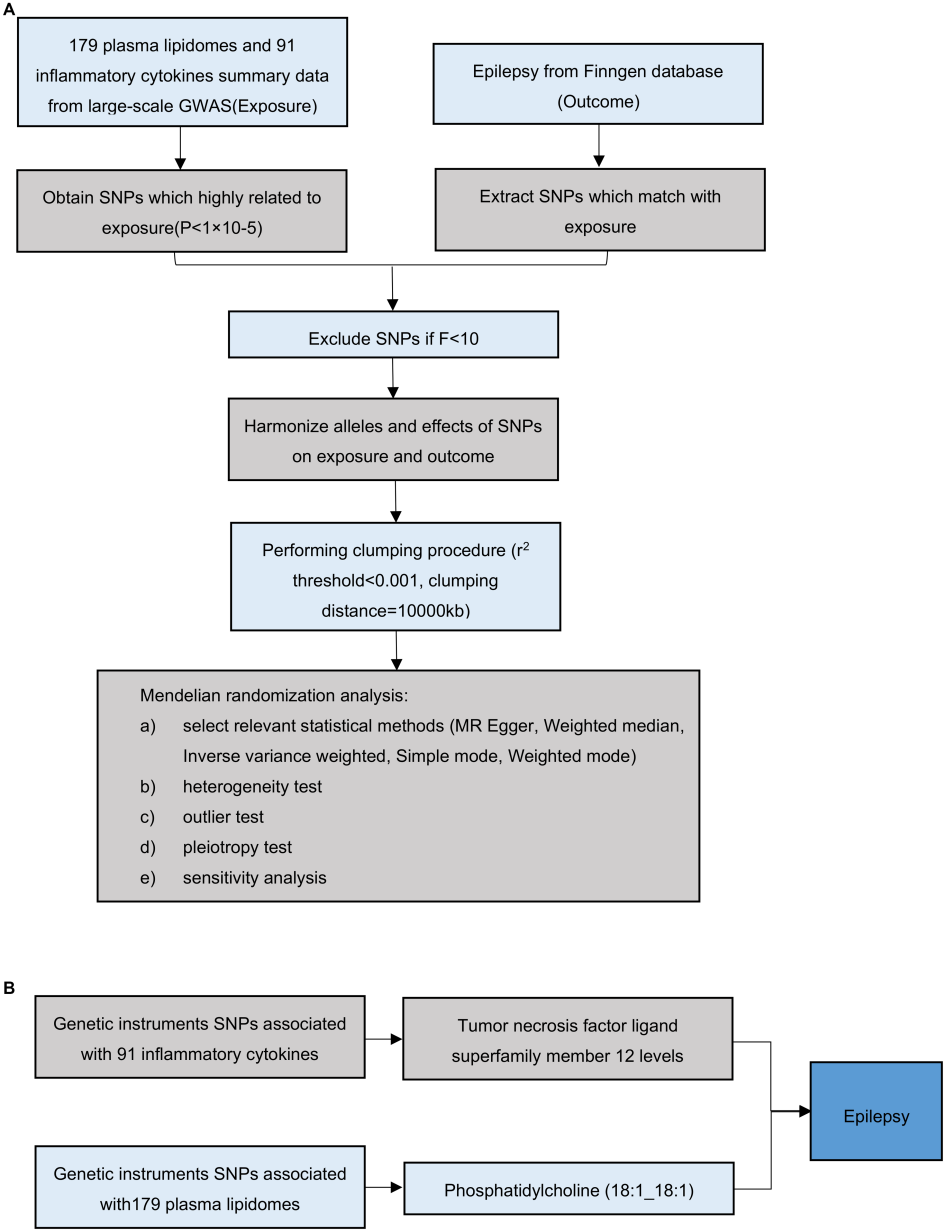
Overall flow chart of this study. **(A)** Illustration of the study design and workflow. **(B)** Two-step Mendelian randomization assessment detailing the impact of Phosphatidylcholine (18:1_18:1) on hypertension through TNFSF12.

The overall flow chart of this study is shown in Figure 1. Using a two-sample MR analysis, we evaluated the causal relationship between 179 plasma lipidomes and 91 inflammatory cytokines in relation to epilepsy. MR uses genetic variation to represent risk factors, necessitating adherence to three hypotheses: (1) instrumental variables (IVs) are closely associated with exposure factors; (2) IVs are unrelated to confounding factors; (3) IVs do not influence outcomes via pathways other than exposure. Subsequently, we employed mediation MR to explore the causal relationship between specific plasma liposomes and epilepsy mediated by inflammatory factors. Our results are reported in accordance with the STROBE-MR guidelines (25). The study included in our analysis obtained approval from the pertinent institutional review committee, with participants having given their informed consent.

### 2.2 GWAS data sources

Regarding exposure variables, we obtained summary statistics on plasma lipidomes through univariate and multivariate GWAS of 179 lipid species from 13 lipid classes in 7,174 Finnish individuals from the GeneRISK cohort. The study conducted a phenome-wide association study (PheWAS) of the identified lipid-associated genetic loci in 377,277 biobank participants of the FinnGen study, followed by a colocalization analysis with these endpoints (accession numbers from GCST90277238 to GCST90277287) (26).

As for mediator phenotypes, we utilized data from genome-wide protein quantitative trait loci (pQTL) mapping for 91 plasma proteins measured using the Olink Target Inflammation panel in 11 cohorts totaling 14,824 European-ancestry participants (accession numbers from GCST90274758 to GCST90274848), (27).

We obtain the GWAS summary-level data on epilepsy from the Finngen_R10.^1^ The study encompassed 325,694 individuals from European, including 12,891 cases of epilepsy and 312,803 controls. Table 1 shows the details of the exposure, mediator and outcome analyzed in our MR study.

**Table 1:** Details of the exposure, mediator and outcome.

Trait	Consortium	Samples	Case	Control
**Exposure**
179 plasma lipidomes	GWAS	7,174	/	/
**Mediator**
91 inflammatory cytokines	GWAS	14,824	/	/
**Outcome**
Epilepsy	Finngen_R10	325,694	12,891	312,803

### 2.3 Selection of instrumental variables

According to recent research (28), Selection of IVs for MR analyses followed specific criteria: (1) Given the minimal number of IVs obtained under the strict threshold (*p* < 5× 10^−8^), a more comprehensive threshold (*p* < 1 × 10^−5^) was adopted to ensure the acquisition of a relatively larger set of IVs for robust results in accordance with the recent study (20); (2) To ensure the independence of each IV, single nucleotide polymorphisms (SNPs) within a window size of 10,000 kb were pruned at a threshold of *r*^2^ < 0.001to mitigate linkage disequilibrium (LD); (3) The *F*-statistic of IVs was calculated to evaluate weak instrumental bias, with an *F*-statistic >10 indicating the absence of bias caused by weak IVs (29).

### 2.4 Statistical analysis

All analyses were conducted using R 4.3.3 software. To assess the causal relationship between plasma lipidomes and epilepsy, inverse variance weighting (IVW) was primarily conducted using the ‘TwoSampleMR’ package (version 0.5.8) (20, 22, 28). Additionally, four other MR methods-MR-Egger, weighted median, simple mode and weighted mode-were employed to complement the final findings. We then used BWMR to further validate our results. Finally, the results of causal associations were presented as odds ratios (OR) and 95% confidence intervals (95% CI). The significance threshold was set at *p* < 0.05.

We conducted a mediation MR analysis employing a two-step MR approach. In the first step, we calculated the causal effect of plasma lipidomes on the mediator (β1), followed by estimating the causal effect of the mediator on epilepsy (β2) in the subsequent step. The delta method was employed to assess the significance of the mediator effect (β1 × β2) and determine the proportion of the mediator effect in the total effect (30).

To assess the stability of causality and evaluate the validity of the hypothesis, we conducted various sensitivity analyses and statistical tests. We used the MR-PRESSO global test and MR-Egger intercept to detect outliers and horizontal pleiotropic effects (31, 32). Furthermore, we conducted a leave-one-out analysis to identify SNPs with potential extreme influence on estimates and to evaluate the robustness of the results. Finally, scatter plots were used to demonstrate that the results were not affected by outliers and funnel plots were used to illustrate the robustness of the correlation and absence of heterogeneity (22). All statistical analyses were performed using the MR and MR-PRESSO packages.

## 3 Results

### 3.1 Instrument variables included in the analysis

Following the previously described criteria for selecting IVs, we were able to obtain exposure IVs from the GWAS database, mediation IVs from the GWAS database and outcome IVs from the Finngen_R10. In our study, all the selected SNPs had F-statistic scores>10 to obtain robust results.

### 3.2 Effects of plasma lipidomes on epilepsy

In our investigation on the effect of plasma liposomes on epilepsy, through MR analysis and BWMR analysis, we identified three plasma liposomes: Phosphatidylcholine (18:1_18:1), Phosphatidylinositol (18:1_18:1) and Sterol ester (27:1/18:0), whose levels were positively correlated with epilepsy. This implies that these lipids may increase the risk of epilepsy (Supplementary Table S1). Conversely, five other plasma lipids, namely Ceramide (d42:1), Sphingomyelin (d38:2), Sphingomyelin (d38:2), Phosphatidylcholine (O-16:1_18:1) and Phosphatidylcholine (18:2_20:1) were linked to a decreased risk of epilepsy, implying potential antiepileptic properties. Following the findings from the MR and BWMR analyses, we chose to further investigate the levels of Phosphatidylcholine (18:1_18:1) for subsequent analyses (OR = 1.105, 95% CI: 1.036–1.178, *p* = 0.002).

**Figure 2:**
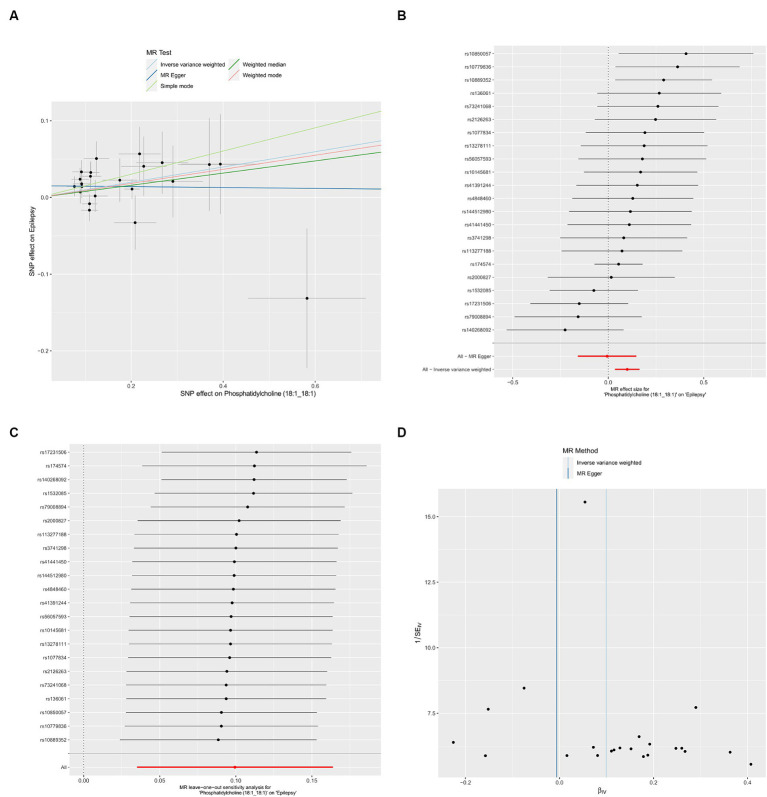
Causal effects of Phosphatidylcholine (18:1_18:1) on epilepsy. **(A)** Scatter plots; **(B)** forest plot; **(C)** leave-one-out analysis; **(D)** funnel plot.

We used IVW method to evaluate the heterogeneity, which indicated that all results exhibited negligible heterogeneity (*p* > 0.05). Additionally, both the MR-Egger intercept test and MR-PRESSO global test indicated that our MR analysis was unaffected by horizontal pleiotropy (*p* > 0.05) (Table 2). Finally, the leave-one-out analysis indicated the robustness of our findings. The Scatter plots, forest plot, leave-one-out analysis and funnel plots illustrating the association of Phosphatidylcholine (18:1_18:1) with epilepsy were presented (Figure 2). Conversely, the reverse MR analyses showed no significant causal effect between Phosphatidylcholine (18:1_18:1) and epilepsy (OR = 0.930, 95% CI: 0.845–1.024, *p* = 0.140) (Supplementary Table S2).

**Table 2:** The results of heterogeneity and horizontal pleiotropy tests between Phosphatidylcholine (18:1_18:1) and epilepsy.

Exposure	Outcome	Heterogeneity test		horizontal pleiotropy test	
		MR Egger regression	IVW model	MR Egger intercept	MR PRESSO test
Phosphatidylcholine (18:1_18:1)	Epilepsy	0.316	0.251	0.156	0.281

### 3.3 Effects of plasma lipidomes on inflammatory cytokines

We conducted additional screening to identify inflammatory cytokines associated with plasma liposomes using a Two-sample MR analysis. The findings are summarized in Supplementary Table S3.

### 3.4 Effects of inflammatory cytokines on epilepsy

**Figure 3:**
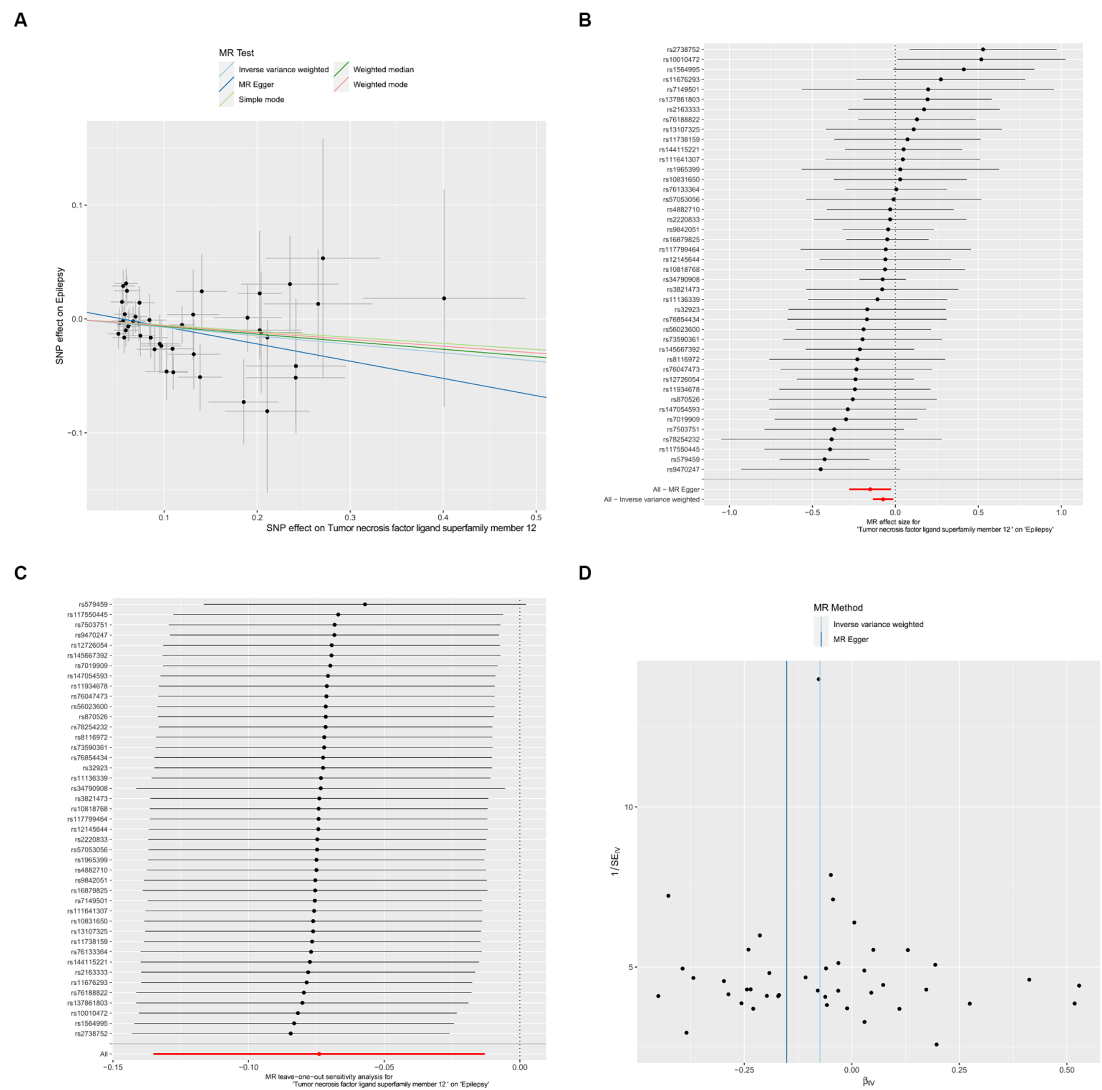
Causal effects of TNFSF12 on epilepsy. **(A)** Scatter plots; **(B)** forest plot; **(C)** leave-one-out analysis; **(D)** funnel plot.

We utilized the inflammatory cytokines identified in the screening for association with plasma liposomes for further analysis. We conducted a Two-sample MR analysis with inflammatory cytokines as the exposure and epilepsy as the outcome. Through our MR analysis, we identified an inflammatory cytokine TNFSF12 associated with epilepsy (OR = 0.929, 95% CI: 0.874–0.987, *p* = 0.018). Scatter plots, forest plot, leave-one-out analysis and funnel plots illustrating the relationship between TNFSF12 and epilepsy were presented (Figure 3).

### 3.5 The mediation effect of TNFSF12 in the causal association between the Phosphatidylcholine (18:1_18:1) and epilepsy

**Figure 4:**
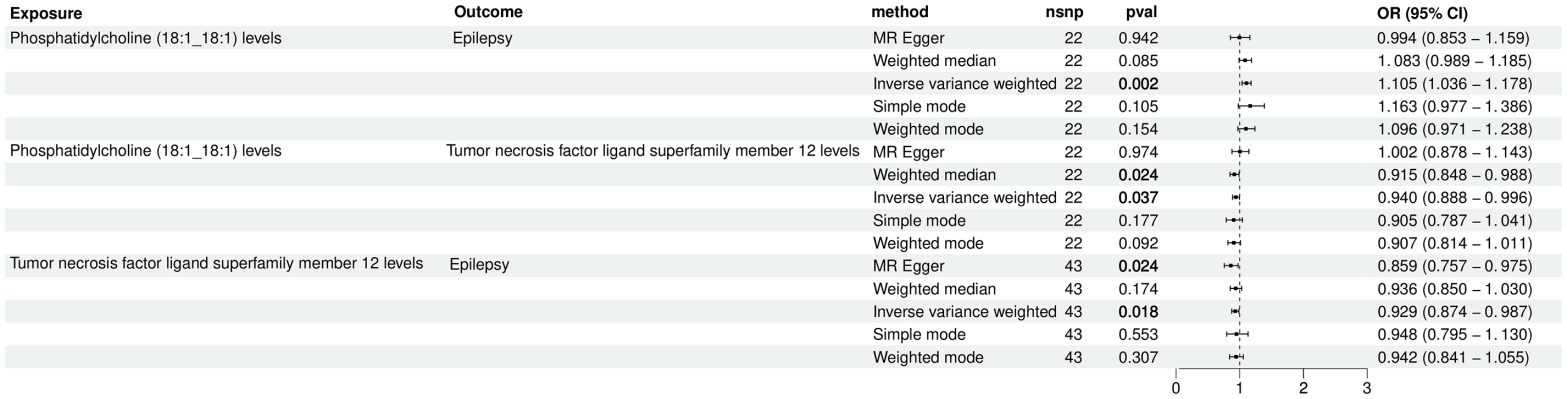
Forest plot for the causal effects of Phosphatidylcholine (18:1_18:1) on epilepsy mediated by TNFSF12.

The two-step MR was employed to perform mediation MR analysis. We aimed to investigate whether the causal relationship between Phosphatidylcholine (18:1_18:1) and epilepsy could be mediated by TNFSF12. Intriguingly, our results revealed that TNFSF12 mediated the causal relationship between Phosphatidylcholine (18:1_18:1) and epilepsy (Figure 4; Table 3). The proportions of mediation were estimated to be 4.58% [mediation effect: (*b* = 0.00455, 95% CI: −0.00120-0.01030), *Z* = 1.552].

**Table 3:** The mediation effect of TNFSF12 on the causal effect of Phosphatidylcholine (18:1_18:1) on epilepsy.

Mediator	Total effect *β*	Direct effect β1	Direct effect β2	Indirect effect β12	*p*	*Z*	Mediation proportion
TNFSF12	0.099	−0.062	0.095	0.005	0.121	1.55	4.58%

## 4 Discussion

The comprehensive mediation MR study was the first to explore the causal relationship between Phosphatidylcholine (18:1_18:1) and epilepsy risk, which revealed a significant causal effect of elevated levels of Phosphatidylcholine (18:1_18:1) on increased susceptibility to epilepsy. Additionally, the results of mediation MR analysis suggest that TNFSF12 may account for 4.58% of the mediating protective effect in the causal pathway from Phosphatidylcholine (18:1_18:1) to epilepsy. This analysis highlights the association between Phosphatidylcholine (18:1_18:1) and epilepsy, emphasizing the mediating role of TNFSF12.

Phosphatidylcholine, an amphoteric molecule, consists of a hydrophilic head and a hydrophobic tail, characterized by a bile base group inserted in the head. Comprising phosphate, choline and fatty acids, it serves as a crucial component of biological membranes and can participate in the transmission of nerve signals (33, 34). Studies indicates that Phosphatidylcholine contributes to brain cell repair, neural connectivity and cognitive enhancement, thereby regulating brain function (35, 36). Cholinergic neurons, through the release of acetylcholine (ACh), modulate neuronal communication, synaptic dynamics, neuroplasticity and hippocampal neurogenesis (37). However, inactivation and imbalance of ACh are associated with central nervous system diseases such as epilepsy. Anticholinergic drugs can block and reduce ACh synaptic activity in the central nervous system, which have been used to treat diseases such as Parkinson’s disease and epilepsy (38, 39). A traumatic brain injury study showed that treatment with anticholinergic drugs in the acute phase after brain injury reduced the frequency and severity of seizures, as well as the number of spontaneous recurrent seizures (40). Animal studies demonstrate that the use of an inhibitory acetylcholine engineered channel activated by exogenous and endogenous agonists can control acute and chronic seizures in male mice (41). Our findings indicate that Phosphatidylcholine (18:1_18:1) could increase the risk of epilepsy, while its reduction could potentially suppress seizures. These findings could provide a promising avenue for future epilepsy research.

TNFSF12, also known as tumor necrosis factor-like weak inducer of apoptosis (TWEAK), is a member of the tumor necrosis factor superfamily implicated in inflammation, apoptosis, autoimmunity and nervous system development (42, 43). Inflammatory cytokines serve as crucial mediators in infection and inflammation, facilitating communication between the brain and the immune system, which are pivotal in epilepsy pathogenesis (14). A study demonstrated that TNFSF12 as a pivotal regulator of astrocyte reactivity, inducing mitochondrial oxidative stress along with protein kinase Cδ and signal transducer and activator of transcription activation (44). This process is accompanied by inflammasome activation and the upregulation and release of proinflammatory cytokines. Astrocytes, pivotal in protecting brain tissue through reactive astrogliosis, exhibit compromised function in epileptic foci, contributing to epileptogenesis (45). Astrocytes in the central nervous system show morphological and functional diversity in brain region-specific patterns. Functional alterations in reactive astrocytes are commonly seen in human temporal lobe epilepsy, while reactive astrocyte-mediated neuroinflammation may contribute to the development of hippocampal epilepsy in animal models (46). Although the precise mechanism of TNFSF12 action in epilepsy remains elusive, our study suggests its potential in reducing epilepsy risk, offering a promising avenue for future clinical investigation.

We conducted the first mediation MR study to explore the causal relationship among plasma liposomes, inflammatory cytokines and epilepsy. Our findings from mediation MR analyses suggest that Phosphatidylcholine (18:1_18:1) may elevate epilepsy risk by reducing TNFSF12 levels, as evidenced by data from large GWAS cohorts utilizing genetic variants. In addition to employing various sensitivity analyses, we accounted for confounding factors and reverse causality, thereby bolstering theoretical underpinnings for epilepsy treatment, prevention and management approaches.

Our study possesses several advantages. Firstly, the sample size is substantial, enhancing the efficacy of the analysis, particularly with the inclusion of large-scale GWAS data. Additionally, we employed various sensitivity analysis techniques to mitigate the influence of confounding factors and reverse causality, thus ensuring the reliability of our causal effect estimates derived from observational studies (47, 48).

The limitations of the present study are as follows: (i) Since the number of IVs fulfilling the strict threshold (*p* < 5 × 10^−8^) was extremely small, a more relaxed threshold (*p* < 1 × 10^−5^) was adopted for screening IVs. (ii) Although the study involved a large sample size, it is crucial to acknowledge that the participants were exclusively of European ancestry, potentially limiting the generalizability of our findings to other regions or ethnicities. (iii) Furthermore, our results remain theoretical and await validation through clinical or animal experiments. Further cellular, animal and clinical investigations are warranted to elucidate these mechanisms.

## 5 Conclusion

Our mediation MR research suggests potential causal relationships among the plasma lipidomes, inflammatory cytokines and epilepsy. Specifically, TNFSF12 mediates the regulatory effect of Phosphatidylcholine (18:1_18:1) on epilepsy. These findings offer genetic evidence of the association between TNFSF12, Phosphatidylcholine (18:1_18:1) and epilepsy. Additionally, our research may facilitate the identification of biochemical markers for predicting, screening and early diagnosing of epilepsy, opening new avenues for exploring its biological mechanisms.

## Data availability statement

The original contributions presented in the study are included in the article/Supplementary material, further inquiries can be directed to the corresponding authors.

## Ethics statement

Ethical review and approval was not required for the study on human participants in accordance with the local legislation and institutional requirements. Written informed consent from the patients/participants or patients/participants’ legal guardian/next of kin was not required to participate in this study in accordance with the national legislation and the institutional requirements.

## Author contributions

XW: Conceptualization, Data curation, Formal analysis, Investigation, Methodology, Project administration, Software, Validation, Visualization, Writing – original draft, Writing – review & editing. WX: Conceptualization, Data curation, Investigation, Methodology, Writing – review & editing. ML: Methodology, Validation, Writing – review & editing. LW: Conceptualization, Software, Writing – review & editing. YZ: Methodology, Project administration, Supervision, Writing – review & editing. CZ: Methodology, Project administration, Writing – review & editing. WL: Formal analysis, Project administration, Resources, Supervision, Validation, Writing – review & editing. SC: Methodology, Project administration, Resources, Visualization, Writing – review & editing. HH: Funding acquisition, Resources, Supervision, Visualization, Writing – review & editing.

## Footnotes

^1^ https://storage.googleapis.com/finngen-public-data-r10/summary_stats/finngen_R10_G6_EPLEPSY.gz
